# Numerical Simulation of Nonlinear Lamb Waves Used in a Thin Plate for Detecting Buried Micro-Cracks

**DOI:** 10.3390/s140508528

**Published:** 2014-05-15

**Authors:** Xiang Wan, Qing Zhang, Guanghua Xu, Peter W. Tse

**Affiliations:** 1 School of Mechanical Engineering, Xi'an Jiaotong University, Xi'an 710049, China; E-Mails: wx72420@163.com (X.W.); xugh@mail.xjtu.edu.cn (G.X.); 2 State Key Laboratory for Manufacturing System Engineering, School of Mechanical Engineering, Xi'an Jiaotong University, Xi'an 710049, China; 3 The Smart Engineering Asset Management Laboratory (SEAM), Department of Systems Engineering and Engineering Management (SEEM), City University of Hong Kong, Tat Chee Avenue, Kowloon, Hong Kong 999077, China; E-Mail: Peter.W.Tse@cityu.edu.hk

**Keywords:** finite element method, simulation, micro-crack, nonlinear Lamb waves, contact acoustic nonlinearity effect, second harmonic, amplitude of second harmonic, amplitude ratio

## Abstract

Compared with conventional linear ultrasonic inspection methods, which are sensitive only to severe defects, nonlinear ultrasonic inspection methods are better for revealing micro-cracks in thin plates. However, most nonlinear ultrasonic inspection methods have only been experimentally investigated using bulk or Rayleigh waves. Numerical studies, especially numerical simulations of Lamb ultrasonic waves, have seldom been reported. In this paper, the interaction between nonlinear S0 mode Lamb waves and micro-cracks of various lengths and widths buried in a thin metallic plate was simulated using the finite element method (FEM). The numerical results indicate that after interacting with a micro-crack, a new wave-packet was generated in addition to the S0 mode wave-packet. The second harmonics of the S0 mode Lamb waves and the new wave-packet were caused by nonlinear acoustic effects at the micro-crack. An amplitude ratio indicator is thus proposed for the early detection of buried micro-cracks.

## 1. Introduction

Cracks can be a major source of concern in safety-critical structures, such as the vital components of aircraft, nuclear power plants, chemical plants and refineries, because they can lead to serious damage or fractures. The use of non-destructive evaluation (NDE) methods for the detection or identification of cracks at early fracture stage, especially incipient buried micro-cracks, is very important for ensuring structural safety and integrity.

The dye penetrant inspection approach is a traditional low-cost NDE tool that has been widely applied for detecting cracks in components used in many mechanical fields, but the technique requires direct access to the specimen and is unable to detect cracks hidden or buried below surfaces. Another popular and powerful NDE tool, the conventional ultrasonic testing method [1,2], has been extensively used to detect and measure the volume of defects such as buried cracks, corrosion, or voids. However, conventional ultrasonic inspection methods using longitudinal or shear waves are time-consuming and inefficient when dealing with large-scale structures because inspection is usually performed in a point-by-point manner. Traditional ultrasonic techniques are also not suitable for detecting cracks perpendicular to the upper and lower surfaces of thin plates. An alternative way to overcome these two limitations is the Lamb wave technique.

In a thin plate, the boundaries of the structure interact with waves, causing successive reflections, refractions and mode conversions that create Lamb waves via a complex mixture of constructive and destructive interference. Compared with traditional ultrasonic longitudinal or shear wave technology, Lamb waves can travel long distances along the structure and can be applied to inspect large areas quickly and efficiently. They have thus been widely used in structural integrity inspection and crack detection in thin structures. The propagation direction of Lamb waves in thin plates is normal to the crack in case of cracks perpendicular to the upper and lower surfaces of the plates, unlike traditional longitudinal waves, for which the propagation direction is parallel to the crack. Lamb waves are thus more suitable for detecting this kind of cracks. Many studies have been conducted on the interaction of Lamb waves with cracks [3–6].

Both the conventional ultrasonic bulk wave inspection method and the Lamb wave technique are based on linear theory, and both depend on measuring particular parameter, such as sound velocity, attenuation, or the transmission and reflection coefficients of the propagating waves. These parameters are sensitive only to gross defects, opened cracks, or macro-cracks within structures. Consequently, linear theory-based ultrasonic NDE methods are unable to detect micro-cracks.

Nonlinear ultrasonic behaviors include nonlinear resonance, mixed frequency response, sub-harmonics generation, and higher harmonics generation. The use of nonlinear technologies has been investigated as an approach to overcome the limitations of linear technologies [7–10]. In this paper, we have investigated higher harmonics generation (mainly second harmonic generation) from a micro-crack. The basic physical mechanism of interest is contact acoustic nonlinearity (CAN) [11], a phenomenon in which a crack caused by longitudinal acoustic traction creates clapping at the crack interface. This clapping nonlinearity originates from the asymmetrical dynamics of the contact stiffness, which is higher in the compression phase than in the tensile stress phase. As a result, the waveform of the acoustic wave is distorted, and higher harmonics are generated in the transmitted wave. Since the pioneering experimental observation of acoustic harmonic generation at fatigue cracks reported by Buck *et al.* [12], there have been a tremendous number of investigations into acoustic harmonic generation, due to its potential application in the detection of cracks [13–15]. However, most studies have used bulk waves or Rayleigh waves, neither of which is suitable for detecting micro-cracks buried in thin structures.

Nonlinear Lamb wave technology is of great interest, because it combines the high sensitivity of the nonlinear approach with the large testing range of Lamb waves, making it a perfect candidate for the detection of micro-cracks hidden in thin materials. Recently, an experimental investigation into the use of nonlinear Lamb waves for evaluating fatigue micro-cracks was carried out [16]. Although in that particular investigation the micro-crack introduced into the inspected specimen was visible and on the surface, it has been experimentally shown that the nonlinear Lamb wave technique has potential for detecting structural micro-cracks. Only a few investigations into this topic have used the finite element method. Kawashima *et al.* [17] studied CAN using Rayleigh waves to detect surface cracks. Soshu and Toshihiko [18] used nonlinear longitudinal waves to detect a closed crack. Recently, Shen and Giurgiutiu [19,20] adopted FEM to simulate the interaction between nonlinear Lamb waves and a surface-breathing crack in a plate. However, their approach had several limitations. First, both the S0 and A0 modes were excited, which means that when the receiving sensor was located close to the crack zone, the S0 and A0 modes received may have been overlapping, and not easily separated. Second, Shen and Giurgiutiu studied a surface breaking-crack, which does not accurately represent most real cracks found in nuclear power, chemical and refinery plant components. Third, the authors only investigated the dependence of the nonlinear effect index on the crack length, and not the crack width.

We used finite element analysis to simulate the interaction between nonlinear Lamb waves and a buried micro-crack perpendicular to the upper and lower surfaces of a thin metallic plate. A pitch and catch approach was applied in the FEM model, using two symmetric piezoelectric ceramic transducer (PZT) wafers as transmitters to generate a single S0 mode signal, and a single PZT wafer as the receiver. A buried, oval-shaped micro-crack was simulated by hard contact with a frictionless model. The generated S0 mode Lamb waves propagated along the structure, interacted with a micro-crack, obtained nonlinear features, and were picked up by the receiver. The interactions between Lamb waves and micro-cracks of different lengths with a constant width were simulated to study the influence of micro-crack length on the nonlinear effects The influence of micro-crack width on nonlinear effects was also investigated by simulating the interaction of Lamb waves with micro-cracks of different widths and a constant length. The simulation results show that the nonlinear Lamb wave technique is indeed capable of detecting a micro-crack in a thin plate. This allows us to propose a baseline-free indicator to identify and detect a micro-crack in a thin plate.

The remainder of this paper is organized as follows: Section 2 introduces the basic theoretical background of Lamb waves and higher harmonics generation through CAN. In Section 3, a finite element model of the interaction between nonlinear Lamb waves and micro-cracks is described in detail. FEM simulation results are presented and discussed in Section 4, and conclusions are drawn and future studies proposed in Section 5.

## 2. Basic Theoretical Background

In this section, we briefly introduce the basic theoretical background related to Lamb wave technology and higher harmonics generation mechanism based on CAN.

### 2.1. Lamb Wave Technology

When stress waves that are excited by a general transducer propagate along a thin structure with key dimensions comparable to the wavelength, they are constrained between its geometric boundaries. Thus, a complex mixture of constructive and destructive interference occurs due to successive reflections, refractions and mode conversions. As a result, Lamb waves are generated. Complications encountered when applying Lamb waves to NDE include the existence of multiple modes and the dispersive behavior of the modes.

#### 2.1.1. Multi-Mode Nature and Dispersive Behavior of Lamb Waves

Classical Lamb waves are defined as elastic waves of plane strain propagating in a traction-free, homogeneous and isotropic plate. The Lamb wave propagation problem is governed by the Navier equation and the boundary conditions of free surface traction. There are two groups of waves, symmetric and anti-symmetric, in which the normal displacement of the particles is symmetric or anti-symmetric, respectively, with respect to the median plane of the plate thickness, that satisfy the wave governing equation and the boundary conditions. Dispersion curves, which plot the phase and group velocities against the excitation frequency, are a fundamental way of describing the propagation of Lamb waves in a specified structure. Dispersion curves can be plotted from the calculated results of the frequency equations for both symmetric and anti-symmetric waves as expressed in Equations (1) and (2) [21]:

(1) $$\frac{\tan qh}{\tan ph}=‐\frac{4pqk^{2}}{{\left(q^{2}‐k^{2}\right)}^{2}},$$

(2) $$\frac{\tan qh}{\tan ph}=‐\frac{{\left(q^{2}‐k^{2}\right)}^{2}}{4pqk^{2}},$$

where 2*h* is the thickness of a plate, and *k* is the wave number. If the longitudinal wave velocity, the shear wave velocity and the angular frequency are denoted by *c_L_, c_T_* and *ω*, then *p* and *q* are described by the following equation [21]:

(3) $$p^{2}=\frac{\omega^{2}}{c_{L}^{2}}-k^{2},q^{2}=\frac{\omega^{2}}{c_{T}^{2}}-k^{2}$$

**Figure 1.**
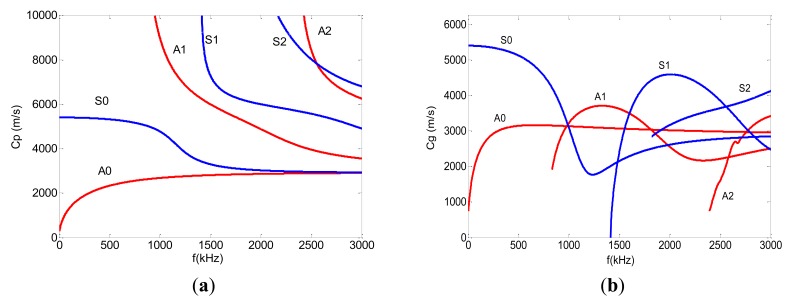
(**a**) Phase velocity dispersion curves for a 2 mm thick aluminum plate, Cp represents phase velocity. (**b**) Group velocity dispersion curves for a 2 mm thick aluminum plate, Cg represents group velocity.

Phase and group velocity dispersion curves for a 2 mm thick aluminum plate are illustrated in Figure 1a,b, respectively. Red curves labeled S0, S1, S2 and blue curves labeled A0, A1, A2 represent the first three symmetric and anti-symmetric mode dispersion curves respectively. At lower frequencies, the velocity of the first symmetric mode (S0) is almost non-dispersive.

Compared to longitudinal or transverse waves whose phase and group velocities are equivalent and independent of frequency, Lamb waves are dispersive in nature, such that the phase and group velocities are not equal, and both velocities are functions of frequency. As the ultrasound energy propagates at the group velocity, the energy of a pulse spreads out in space and time as it progresses through a material.

The multi-mode and dispersive nature of Lamb waves creates difficulties in the interpreting the signals received. Thus, it is desirable to generate a single mode to use Lamb waves in NDE applications.

The S0 mode at low frequencies is promising in NDE for three reasons. First, it is practically non-dispersive. Second, its stresses are almost uniform through the thickness of the plate so that its sensitivity to defects is not dependent on the thickness of the defect location [22]. Third, it is the fastest mode, which indicates that it will be the first wave-packet to arrive at the receiver, and so can readily be separated from other signals by time domain gating.

#### 2.1.2. Single S0 Mode Excitation

**Figure 2.**
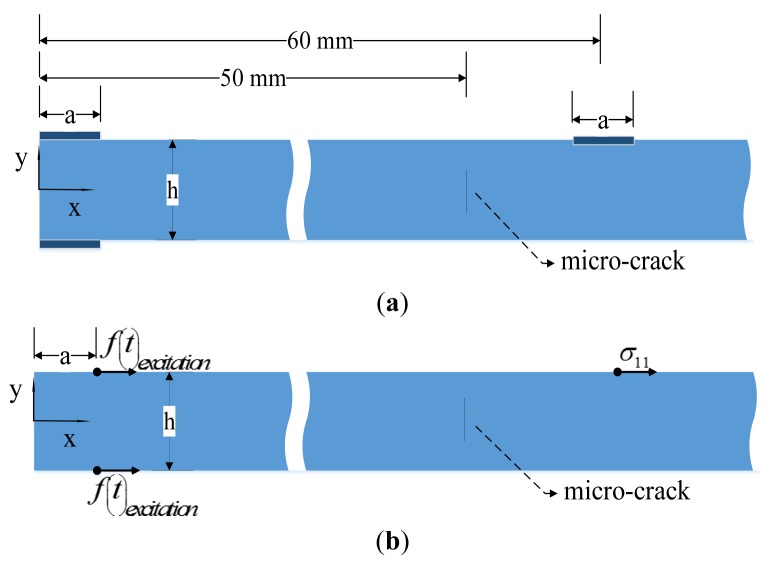
(**a**) Geometry of the plate for simulations and PZT wafer's configuration. (**b**) Point force model for simulations.

Figure 1 shows that there are at least two modes for any chosen frequency. This means that selectively exciting one mode is difficult. Several approaches, including angled prisms [23], comb transducers [24,25] and linear arrays with time-delayed excitation [26,27], have been developed to excite single-mode Lamb waves. Recently, PZT wafers have been explored as transmitters to excite and sensors to detect Lamb waves. A theoretical explanation for the mode selectivity of the PZT wafer transducer has been reported in [28]. In our paper, three PZT wafers were bonded on a 2 mm thick aluminum plate. Two PZT wafers used as actuators were placed on the double surface at the excitation point, meaning that the two transmitters had the same coordinates. When these two PZT actuators were excited by the same input burst signal, symmetric mode Lamb waves were enhanced, while anti-symmetric waves were suppressed. Only S0 mode Lamb waves were generated. Another PZT wafer was placed as a receiver to collect the wave signal. The configuration of these transducers is shown in Figure 2a. In our simulation model, the point force method was used to model PZT actuators. This kind of point force modeling is described in detail by Nieuwenhuis *et al.* [29]. Figure 2b illustrates a point force simulation model, in which the principal effect of two PZT actuators are represented as two point forces acting in the x-direction on the upper and lower surface respectively at position x = a (where a = 3.2 mm, the length of a PZT transducer). The PZT receiver is modeled by a point on the upper surface located 60 mm from the y axis, and the stress *σ*_11_ at this point is monitored as the receiving signal.

### 2.2. Higher Harmonics Generation through Contact Acoustic Nonlinearity

When an ultrasonic wave excited by a large amplitude is incident to an imperfect interface, higher harmonic waves are generated. This phenomenon is known as CAN, and has attracted increasing amounts of attention for its potential to characterize closed cracks or imperfect bond interfaces.

**Figure 3.**
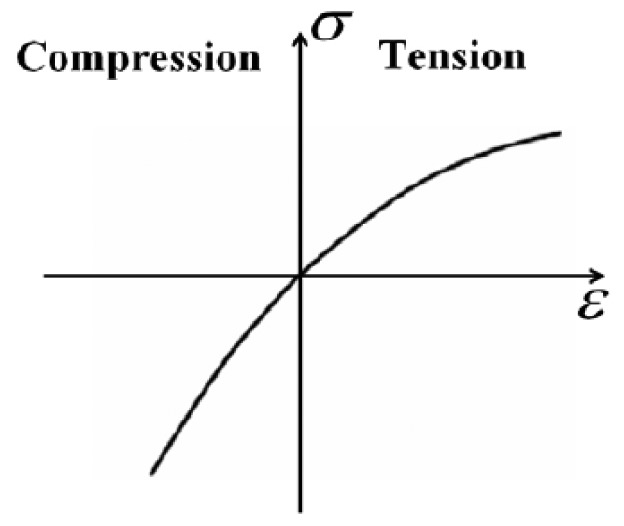
The nonlinear relationship between stress *σ* and strain *ε* [30].

Physically, the phenomenon of higher harmonics generation is related to nonlinearity in the elastic behavior of the material, which indicates that the relationship between stress *σ* and strain *ε* is nonlinear, as illustrated in Figure 3 [30]. This nonlinear relationship can be expressed by the nonlinear version of Hooke's law shown in Equation (4) [31]:

(4) $$\sigma =Eɛ(1+\beta ɛ+\gamma {ɛ}^{2}+\cdots )$$

where *E* is Young's modulus, and *β* and *γ* are second and third order nonlinear elastic coefficients respectively. Here, we consider a nonlinear dynamic system of the form:

(5) $$y=Cu(1+\beta u+\gamma u^{2}+\cdots )$$

where *u* and *y* are the general input and output, respectively, and *C* is a scale factor. Consider a harmonic input:

(6) $$u(\omega )=ũe^{j\omega t}$$

By substituting Equations (6) into (5), the output takes the following form:

(7) $$y=Cu(\omega )+C\beta ũ\cdot u(2\omega )+C\gamma {ũ}^{2}\cdot u(3\omega )+\cdots$$

Equation (7) indicates that the output of the nonlinear system contains not only the fundamental frequency *ω* but also higher order harmonics 2*ω*, 3*ω*…. This distinctive feature makes it possible for us to evaluate the material degradation, assess fatigue, or detect micro-cracks that introduce nonlinearity to the specimen.

**Figure 4.**
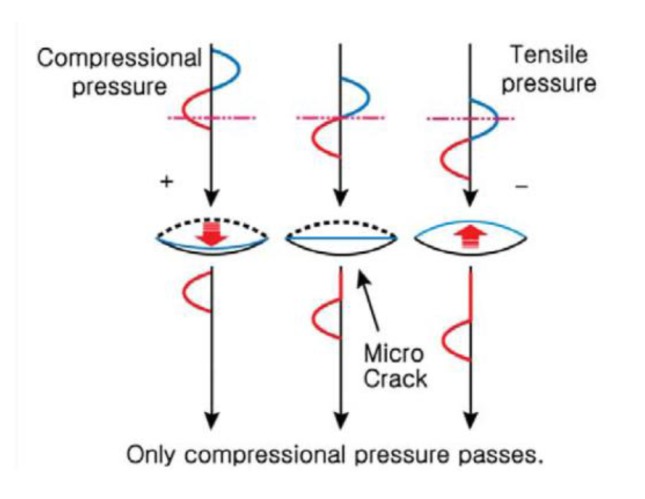
Schematic diagram illustrating the CAN concept of at a micro-crack; only the compressional phase of the ultrasonic wave can penetrate the interface of the crack, whereas the tensile phase cannot.

The basic physical mechanism of CAN is that a crack driven by longitudinal acoustic traction causes clapping of the crack interface. This clapping nonlinearity originates from asymmetrical dynamics of the contact stiffness which is higher in the compression phase than in the tensile phase. As a result, the compressional part of the waves can penetrate it, but their tensile part cannot, as shown in Figure 4 [31]. Therefore, after penetrating the interface, the waves exhibit half-wave rectification, which means that they have obvious nonlinearity. This nonlinearity can then be detected by higher harmonics [30].

## 3. Finite Element Model

Two-dimensional FEM models were developed and dynamic simulations were performed using Abaqus/Explicit software. The parameters of the plate are shown in Table 1.

**Figure 5.**
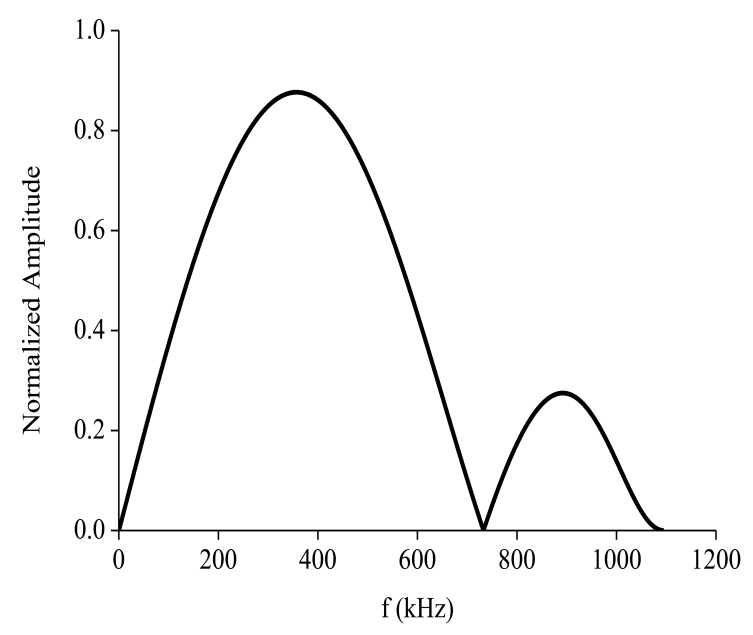
Tuning curve showing that the amplitude of the S0 mode reaches its peak at around 400 kHz.

**Figure 6.**
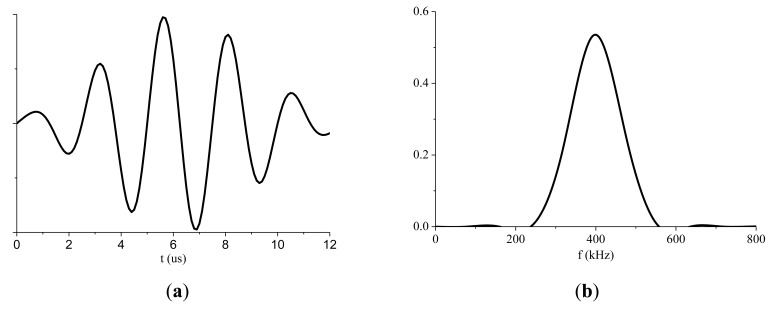
(**a**) Temporal waveform of the excited tone burst signal. (**b**) Frequency spectrum of the excited tone burst signal.

S0 mode Lamb waves display an almost non-dispersive character at low frequencies. The tuning curve was used to select the best centered excitation frequency. The tuning curve is plotted by the WaveFormRevealer software [32,33] developed by LAMSS at the University of South Carolina. As shown in Figure 5, the amplitude of the S0 mode peaks at around 400 kHz. Therefore, the centered excitation frequency was set to 400 kHz. A hamming windowed tone-burst consisting of five cycles at a frequency of 400 kHz was used as the excitation signal. Its mathematical formula is given in Equation (8). The temporal waveform and its corresponding frequency spectrum are plotted in Figure 6a,b:

(8) f(t)=sin(2π⋅400000t)⋅(0.08+0.46(1−cos(2π⋅400000t/5))).

**Figure 7.**
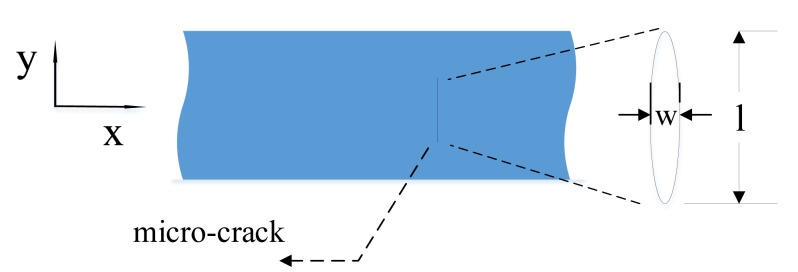
Elliptical shape of the modeled micro-crack.

The micro-crack was located 50 mm from the y axis, and its shape was modeled as an ellipse as shown in Figure 7, and its surfaces were simulated by hard contact with a frictionless model. We define the major and minor axis of the ellipse as the length and width of the crack, respectively.

To obtain adequate accuracy and high efficiency, a meshing strategy with varying mesh density was adopted. In general, a denser mesh will give a more accurate result, but will also cost more in terms of calculation time and computer resources. We adopted the maximum element size and time step to ensure accuracy from the reference [19]:

(9) $$I_{\max}=\frac{\lambda_{\min}}{20}$$

(10) $$\Delta t_{\max}=\frac{1}{20f_{\max}}$$

**Figure 8.**
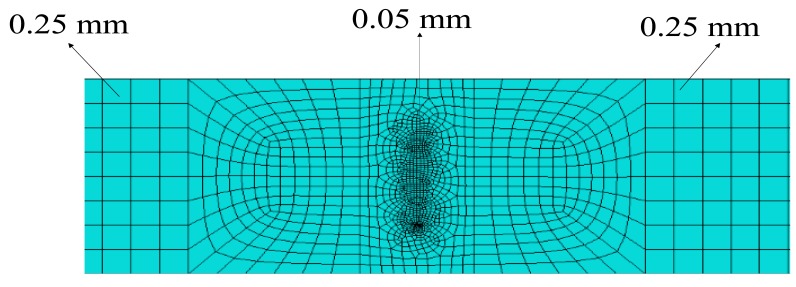
Mesh size in different regions: for the region between the actuator and the crack and, the zone between the crack and the receiver, the mesh size was 0.25 mm, for the cracked area the mesh size was very small equaling to 0.05 mm.

For a 400 kHz signal, according to Equations (9) and (10), the calculated maximum element size and time step are 0.655 mm and 0.125 μs. Therefore, a mesh size of 0.5 mm and a time step of 0.1 μs are sufficient to ensure accuracy. The crack zone was more densely meshed, with much smaller elements to accommodate the complicated mechanical response. In this paper, the element size at the micro-crack was set to 0.05 mm. To ensure the accuracy of the second harmonic generated, a mesh size of 0.25 mm was applied to both the region between the actuators and the micro-crack and the zone between the crack zone and the receiver. The meshing result is depicted in Figure 8. A time step of 0.05 μs was used to ensure the accuracy of the second harmonic.

The initial simulations were carried out for an undamaged plate and a plate with a micro-crack 6 nm wide and 800 μm long, to identify the second harmonic generation features and characteristics of the signals received as a result of the micro-crack. Next, two groups of simulations were performed to investigate the dependence of nonlinear effects on the micro-crack's length and width.

The first group of simulations featured a micro-crack of a constant width and varied length. Width w was set to 6 nm, and length l was set to 200, 400, 600, 800, 1000, 1200 and 1400 μm. We used an index s = l/h (where l and h are the crack length and plate thickness) to define the length severity of the micro-crack. Accordingly, the micro-crack length severity index s was equal to 0.1, 0.2, 0.3, 0.4, 0.5, 0.6 and 0.7. The second group of simulations used a micro-crack of a fixed length and changing width. The value of e length l was fixed to 800 μm, and the values of width w used were 4 nm, 6 nm, 8 nm, 10 nm, 12 nm, 14 nm, 16 nm, 20 nm, 30 nm and 40 nm.

## 4. Simulation Results and Discussions

In this section, we first present and discuss the simulation results generated from both the undamaged plate and the plate with a micro-crack 6 nm wide and 800 μm long. Generated second harmonics in the received wave-packets indicated the existence of a micro-crack in the plate. Then, we display and discuss two groups of simulation results to identify the influence of the micro-crack's length and width on the nonlinear effects. Finally, based on these simulation results and discussions, we propose a baseline free indicator to identify and detect a buried micro-crack in a thin plate.

### 4.1. Second Harmonic Generation as a Result of the Presence of a Micro-Crack

**Figure 9.**
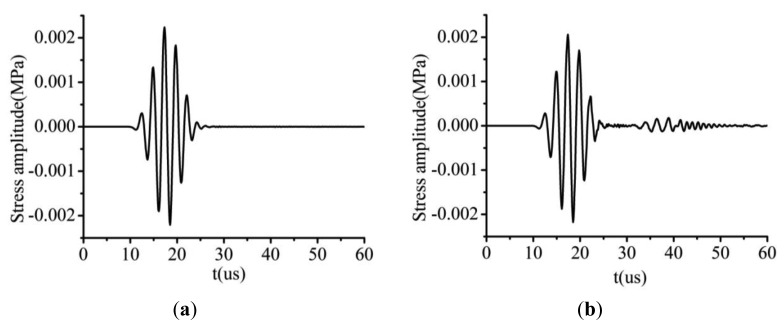
(**a**) Time-domain signal received from an undamaged plate. (**b**) Time-domain signal received from the damaged plate with a micro-crack 6 nm wide and 800 μm long.

**Figure 10.**
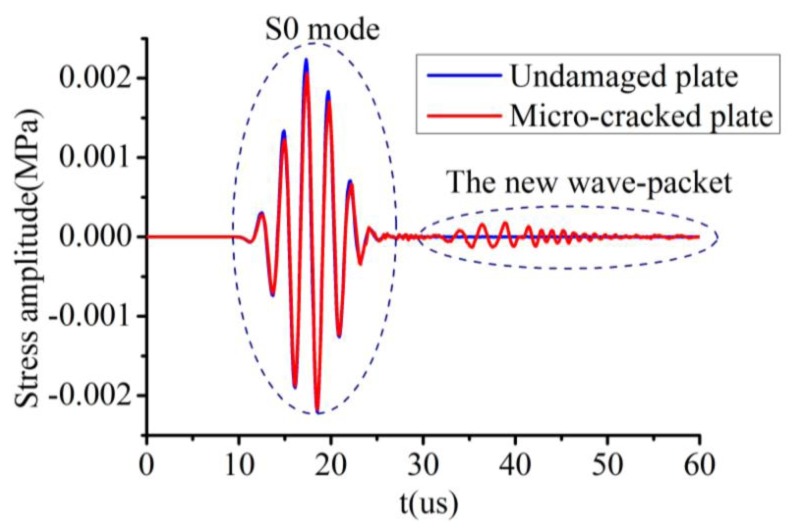
Superimposed time-domain signals. The blue curve represents the signal from the undamaged plate, and the red curve represents the signal from the plate with a micro-crack 6 nm wide and 800 μm long.

**Figure 11.**
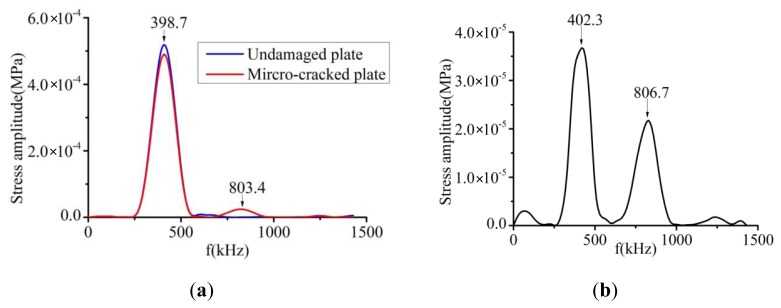
(**a**) Frequency spectra of S0 mode wave signals. The blue curve represents the Fourier spectrum of the S0 mode wave signal from the undamaged plate, and the red curve shows the Fourier spectrum of the S0 mode wave signal from the plate with a micro-crack 6 nm wide and 800 μm long. (**b**) Fourier spectrum of the new wave-packet signal from the damaged plate.

The received time-domain signals from an undamaged plate and a damaged plate with a micro-crack 6 nm wide and 800 μm long are illustrated in Figure 9a,b. To show the differences between these two signals, they are superimposed in Figure 10. It can be clearly seen that a new wave-packet has appeared in the time-domain signal obtained from the micro-cracked plate. Another difference between the signals from the two plates is that there was a slight amplitude drop and phase shift in the S0 mode wave-packet signal from the micro-cracked plate compared with the signal from the undamaged plate. Second harmonic component generated by the nonlinear effect at the micro-crack was introduced into both the S0 mode wave-packet and the new wave-packet. This inference can be confirmed by analyzing the frequency-domain spectrum. Fourier transforms of the S0 mode wave-packets from both the undamaged and the micro-cracked plates were conducted. Their corresponding frequency spectra are plotted in Figure 11a. The frequency spectrum of the new wave-packet signal from the damaged plate is given in Figure 11b.

As indicated in the Fourier spectrum of the S0 mode shown in Figure 11a, two amplitude peaks were present at around 398.7 kHz and 803.4 kHz for the micro-cracked plate, but only one peak appeared at around 398.7 kHz for the undamaged plate. Because the excitation frequency was centered at f = 400 kHz, the 398.7 kHz peak corresponded to the amplitude of the fundamental frequency component, and the 803.4 kHz peak corresponded to the amplitude of the second harmonic component. Hence, for the undamaged plate, no higher harmonic components existed in the received S0 mode wave-packet, whereas a second harmonic component was present in the S0 mode wave-packet of the signal from the micro-cracked plate. Regarding the Fourier spectrum of the new wave-packet signal depicted in Figure 11b, the first amplitude peak presented at 402.3 kHz corresponded to the fundamental frequency component and the second amplitude peak observed at 806.7 kHz corresponded to the second harmonic component. In the new wave-packet, the amplitude of the second harmonic was much more obvious than that of the S0 mode wave-packet. The Fourier spectrum analysis confirmed that the S0 mode wave-packet received from the micro-cracked plate contained a second harmonic component, which was introduced by the micro-crack.

**Figure 12.**
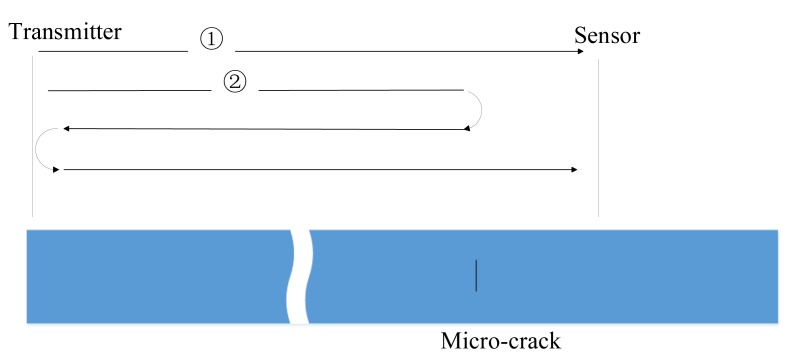
The propagation wave path for the time-domain signal received from the micro-cracked plate with a micro-crack 6 nm wide and 800 μm long.

The propagation wave paths for the time-domain signal received from the micro-cracked plate with a micro-crack 6 nm wide and 800 μm long are illustrated in Figure 12. The propagation wave paths for S0 mode wave-packet and the new wave-packet are shown as path ➀ and ➁ respectively. The S0 mode wave-packet is the directed wave-packet, propagating through and interacting with the micro-crack and received by the sensor. A very small part of directed wave-packet is reflected by the micro-crack, propagating back and reflected by the left end, then propagating forward through and interacting with the micro-crack and finally obtained by the sensor. This is the generation process of the new wave-packet.

The presence of second harmonic components in both the S0 wave-packet and new wave-packet can be used as indicators to detect and identify the existence of a micro-crack in a plate. In addition to the amplitude of the second harmonic components, we used the amplitude ratio of the second harmonic signal (A2) to the fundamental frequency signal (A1) as a nonlinear index to describe the degree of the nonlinear effect shown in Equation (11):

(11) $$\text{Amplitude Ratio}=\frac{A2}{A1}$$

### 4.2. Dependence of Nonlinear Effect on the Micro-Crack's Length

**Figure 13.**
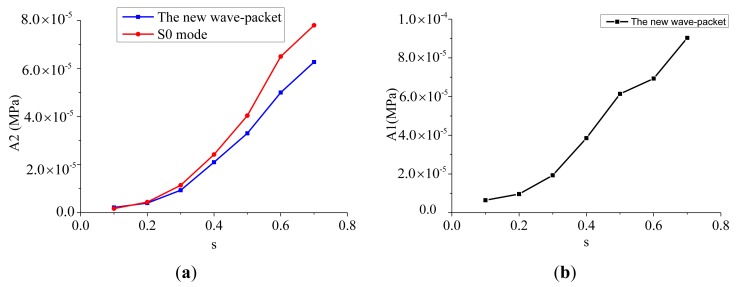
(**a**) Relationship between the amplitude of the second harmonic component and the micro-crack length severity for both the S0 mode wave-packet and the new wave-packet. The red curve represents the relationship for the S0 mode wave-packet, and the blue curve represents the relationship for the new wave-packet; A2 represents the amplitude of the second harmonic component; s represents the micro-crack length severity. (**b**) The relationship between the amplitude of the fundamental frequency component and the micro-crack length severity for the new wave-packet. A1 represents the amplitude of the fundamental frequency component; s represents the micro-crack length severity.

The amplitudes of the second harmonics for both the S0 mode and the new wave-packet at different micro-crack length severities are shown in Figure 13a. It is clear that although the amplitude of the second harmonic is very small, it has a monotonically increasing relationship with the micro-crack length severity for both the S0 wave and the new wave-packet. The longer the micro-crack, the larger the amplitude of the second harmonic becomes. These simulation results are in accordance with the simulation and experimental results reported by Soshu and Toshihiko [18], who used nonlinear longitudinal waves to detect a closed crack. A straightforward explanation is that the contact acoustic nonlinearity which was detected by high harmonics increased with the length of the micro-crack. Therefore, the amplitude of the second harmonics in the S0 mode wave packet and in the new wave packet showed a positive relationship with the length of the micro-crack, as the interaction between the micro-crack and the Lamb waves introduced contact acoustic nonlinearity to both the S0 mode wave-packet and the new wave-packet. As the micro-crack became longer, the contact stiffness of the interface decreased. According to the theory of Buck *et al.* [12] and Biwa *et al.* [34], as the contact stiffness of the interface decreased, that is, as the micro-crack became longer, the acoustic nonlinearity increased. The increased amplitude of the second harmonic components with the length of the micro-crack conformed with this theory.

**Figure 14.**
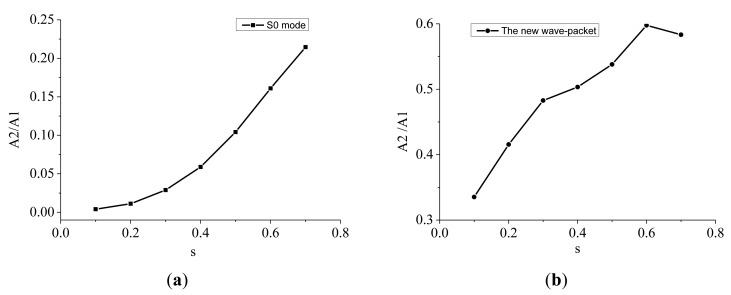
(**a**) Variation of amplitude ratio with the micro-crack length severity for the S0 mode wave-packet. A2/A1 represents the amplitude ratio; s represents the micro-crack length severity. (**b**) The variation of amplitude ratio with micro-crack length severity for the new wave-packet. A2/A1 represents the amplitude ratio; s represents the micro-crack length severity.

The variation of amplitude ratio with micro-crack length severity for the S0 mode wave-packet and the new wave-packet is shown in Figure 14a,b. It can be clearly seen that the amplitude ratio was relatively small for the S0 mode wave-packet compared with the new wave-packet, and that the amplitude ratio for the former wave-packet also increased monotonically with the micro-crack length severity, In contrast, the amplitude ratio for the new wave-packet increased monotonically to a peak value and then decreased. Shen and Giurgiutiu [19] obtained similar results through simulating S0 and A0 mode Lamb waves simultaneously interacting with a surface-breathing crack. The amplitude ratio for the new wave-packet was much larger than that for the S0 mode wave-packet, which is attributed to the fact that the amplitude of the fundamental frequency component of the S0 mode wave-packet was much larger than that of the new wave-packet. As the amplitude changes of the fundamental frequency component of the S0 mode wave-packet from seven micro-cracked plates were very small, the variation of the amplitude ratio with micro-crack length severity had the same increasing trend as the amplitude of the second harmonic component of the S0 mode wave-packet.

The amplitude of fundamental frequency component also had a monotonically increasing relationship with the micro-crack length severity, as shown in Figure 13b. The amplitude of the fundamental frequency component of the new wave-packet increased faster than that of the second harmonic component at large micro-crack length severities, which caused the amplitude ratio for the new wave-packet to drop. The amplitude ratio for the new wave-packet was relatively large, even for short-length micro-cracks. This ratio can thus be used as an index to identify micro-cracks with small lengths.

### 4.3. Dependence of Nonlinear Effect on the Micro-Crack's Width

**Figure 15.**
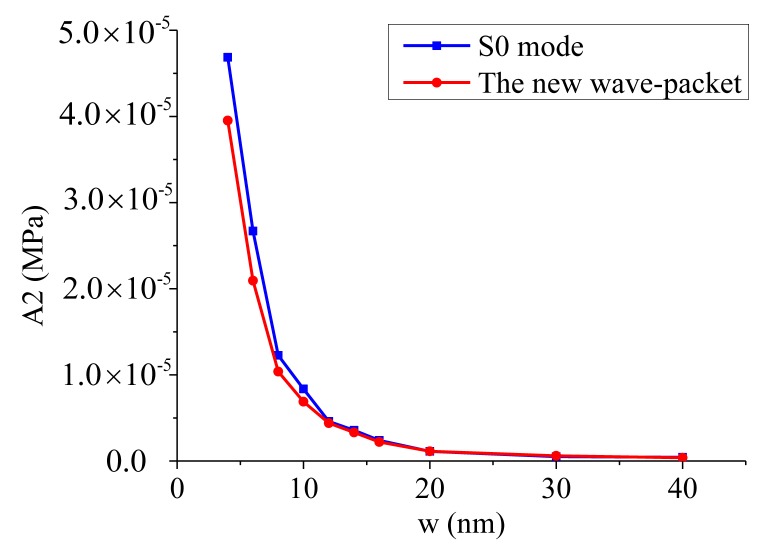
Relationship between the amplitude of the second harmonic component and the width of a micro-crack for both the S0 mode wave-packet and the new wave-packet. The blue curve represents the relationship for the S0 mode wave-packet, and the red curve represents the relationship for the new wave-packet. A2 represents the amplitude of the second harmonic; w represents the width of a micro-crack.

**Figure 16.**
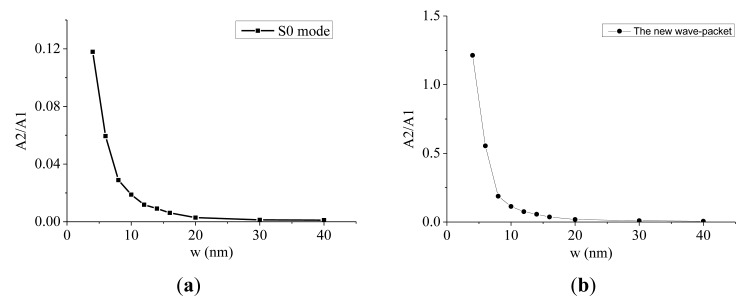
(**a**) The variation of the amplitude ratio with the width of a micro-crack for the S0 mode wave-packet. A2/A1 represents the amplitude ratio; w represents the width of a micro-crack. (**b**) The variation of the amplitude ratio with the width of a micro-crack for the new wave-packet. A2/A1 represents the amplitude ratio; w represents the width of a micro-crack.

The amplitudes of the second harmonics for both the S0 mode and the new wave-packet at different micro-crack widths are shown in Figure 15. In contrast to the positive relationship between the amplitude of the second harmonic component and the length of the micro-crack, the amplitude of the second harmonic had a monotonically decreasing relationship with the width of the micro-crack for both the S0 mode wave-packet and the new wave-packet. The wider the micro-crack was, the smaller the amplitude of the second harmonic became. Moreover, the amplitude ratio decreased as the micro-crack became wider for both the S0 mode wave-packet and the new wave-packet, as shown in Figure 16a,b. As the micro-crack became wider, the gap between its two interfaces became larger. Consequently, some areas of the interfaces were not in contact during the compressional phase of the incident wave, and thus the contact area was reduced. As a result, the amplitudes of the second harmonic components were also reduced. From these Figures 15 and 16, we can see that the amplitude ratio and the amplitude of the second harmonic components for both the S0 mode wave-packet and the new wave-packet dropped very quickly when the width of the micro-crack was less than 10 nm. When the width exceeded 20 mm, the amplitude ratio and second harmonic components for both the S0 mode wave-packet and the new wave packet reached a very low saturation level. The amplitude of the second harmonic components and the amplitude ratio for both the S0 mode wave-packet and the new wave-packet were very sensitive to micro-cracks with small widths. These measures can thus be used to detect and identify small-width micro-cracks. However, the amplitudes of the second harmonic components for both the S0 mode wave-packet and the new wave-packet were very small, and the amplitude ratio for the S0 mode was also relatively small compared with that of the new wave-packet. Thus these measures may not be so sensitive in high-noise conditions. However, the amplitude ratio for the new wave-packet is relatively large and can be used to detect and identify micro-cracks with small widths.

After analyzing the effect of the micro-crack's length and width on nonlinear effects, the amplitude ratio for the new wave-packet could serve as an early indicator of a buried micro-crack, because the ratio is sensitive enough to detect micro-cracks with small lengths and small widths. In addition, as this method depends only on the ratio of Fourier spectrum amplitudes, it does not require any baseline data. Therefore, the amplitude ratio for the new wave-packet is a baseline-free indicator of a micro-crack presence. However, in practical experiments, higher harmonic components will also induced by inherent nonlinearity of electrical equipment (e.g., signal generator, amplifier) which should be removed first before using the amplitude ratio of the new wave-packet as a baseline-free indicator [35].

## 4. Conclusions

The interaction between nonlinear single S0 mode Lamb waves and a buried micro-crack in a thin plate were simulated using FEM. First, a finite element model was established using the Abaqus software. The point force method was applied to approximate PZT wafer transmitters; the micro-crack was modeled as an oval shape with hard contact and frictionless surfaces; an optimal exciting centered frequency was selected via a tuning curve, and proper element size and time step were selected to ensure the model's accuracy and efficiency. Second, simulations of an undamaged plate and a plate with micro-cracks of different lengths and widths were then carried out. Finally, a baseline-free indicator for the detection of a micro-crack buried in a thin plate was proposed.

The simulation results showed that a new wave-packet appeared in the received temporal signal when a micro-crack was present. Fourier spectrum analysis revealed no higher harmonics in the S0 mode wave-packet received from the undamaged plate. However, second harmonics introduced by contact acoustic nonlinearity at the micro-crack could be clearly observed in both the S0 mode and the new wave-packet signal from the micro-cracked plate. We also investigated the dependence of nonlinear effects on the length and width of the micro-crack. The amplitude of the second harmonic components for both the S0 mode wave-packet and the new wave-packet, and also the amplitude ratio for the S0 mode wave-packet, showed a monotonic increasing relationship with the length of the micro-crack. However, the amplitude ratio for the new wave-packet increased with the length of the micro-crack to a peak, and then decreased. The amplitude ratio for the new wave-packet was relatively large, even for short micro-cracks. The ratio can thus be used as an index for detecting a short-length micro-crack. The amplitude of the second harmonic components and the amplitude ratio for both the S0 mode wave-packet and the new wave-packet had the same decreasing relationship with the width of the micro-crack. A steep relationship was shown when the width of the micro-crack was less than 10 nm, and thus these measures are highly sensitive to micro-cracks with very small widths. As the amplitude ratio for the new wave-packet was relatively large, this ratio could be more reliable and sensitive in high-noise environments. Therefore, the amplitude ratio for the new wave-packet can be used as an early indicator of a buried micro-crack presence. This paper makes three contributions to the literature. First, it provides a finite element model of the interaction between nonlinear single S0 mode Lamb waves and a micro-crack buried in a thin plate. Second, it demonstrates the dependence of nonlinear effects on both the length and width of the micro-crack. Third, it proposes the amplitude ratio for the new wave-packet as a baseline-free early indicator for buried micro-cracks in a thin structure. This paper has only focused on situations involving a single micro-crack, but further investigations will concentrate on plates with multiple micro-cracks.

## Figures and Tables

**Table 1.** The parameter of the plate.

**Thickness (h)**	**Density (**ρ**)**	**Elasticity Modulus (E)**	**Poisson's Ratio (*λ*)**
2 *mm*	2700 *kg/m^3^*	69 *GPa*	0.33

## References

[1] Yang X.X., Chen S.L., Jin S.J., Chang W.S. (2013). Crack orientation and depth estimation in a low-pressure turbine disc using a phased array ultrasonic transducer and an artificial neural network. Sensors.

[2] Jeanne L.S., Kuo T.W., Cheng K.J., Chun H.C., Jing C.T., Jiunn W.L. (2013). Applications of flexible ultrasonic transducer array for defect detection at 150 °C. Sensors.

[3] Castaings M., Le C.E., Hosten B. (2002). Modal decomposition method for modeling the interaction of Lamb waves with cracks. J. Acoust. Soc. Am..

[4] Le C.E., Castaings M., Hosten B. (2002). The interaction of the S0 Lamb mode with vertical cracks in an aluminum plate. Ultrasonics.

[5] Wang L.G., Shen J.Z. (1997). Scattering of elastic waves by a crack in an isotropic plate. Ultrasonics.

[6] Lu Y., Ye L., Su Z.Q., Yang C.H. (2008). Quantitative assessment of through-thickness crack size based on Lamb wave scattering in aluminium plates. NDT E Int..

[7] Abeelel K., Windels F., Paolo P. (2006). Characterization and imaging of microdamage using nonlinear resonance ultrasound spectroscopy (NRUS): An analytical model. Universality of Nonclassical Nonlinearity Part III.

[8] Ulrich T.J., Sutin A.M., Guyer R.A., Johnson P.A. (2008). Time reversal and nonlinear elastic wave spectroscopy (TR–NEWS) techniques. Int. J. Nonlinear Mech..

[9] Solodov I., Pfleiderer K., Busse G., Paolo P. (2006). Nonlinear acoustic NDE: Inherent potential of complete nonclassical spectra. Universality of Nonclassical Nonlinearity Part III.

[10] Jhang K.Y. (2000). Applications of nonlinear ultrasonics to the NDE of material degradation. IEEE Trans. Ultrason. Ferroelectr. Freq. Control.

[11] Lee T.H., Jhang K.Y. (2009). Experimental investigation of nonlinear acoustic effect at crack. NDT E Int..

[12] Buck O., Morris W.L., Richardson J.M. (1978). Acoustic harmonic generation at unbonded interfaces and fatigue cracks. Appl. Phys. Lett..

[13] Nazarov V.E., Sutin A. (1997). Nonlinear elastic contacts of solids with cracks. J. Acoust. Soc. Am..

[14] Solodov I.Y., Korshak B.A. (2002). Instability, chaos, and “memory” in acoustic-wave-crack Interaction. Phys. Rev. Lett..

[15] Rokhlin S.I., Kim J.Y. (2003). *In situ* ultrasonic monitoring of surface fatigue crack initiation and growth from surface cavity. Int. J. Fatigue.

[16] Dutta D., Sohn H., Harries K.A. (2009). A nonlinear acoustic technique for crack detection in metallic structures. Struct. Health Monit..

[17] Kawashima K., Omote R., Ito T., Fujita H., Shima T. (2002). Nonlinear acoustic response through minute surface cracks: FEM simulation and experimentation. Ultrasonics.

[18] Soshu H., Toshihiko S., Thompson D.O. (2006). Detection of a closed crack by nonlinear acoustics using ultrasonic transducers. Review of Progress in Quantitative Nondestructive Evaluation.

[19] Shen Y., Giurgiutiu V. Predictive simulation of nonlinear ultrasonics.

[20] Shen Y., Giurgiutiu V. (2014). Predictive modeling of nonlinear wave propagation for structural health monitoring with piezoelectric wafer active sensors. J. Intell. Mater. Syst. Struct..

[21] Achenbach J.D. (1973). Wave Propagation in Elastic Solids.

[22] Lowe M.J.S., Diligent O. (2002). Low-frequency reflection characteristics of the S0 Lamb wave from a rectangular notch in a plate. J. Acoust. Soc. Am..

[23] Rose J.L. (2000). Guided wave nuances for ultrasonic nondestructive evaluation. IEEE Trans. Ultrason. Ferroelec. Freq. Control.

[24] Quarry M.J., Rose J.L. (1999). Multimode guided wave inspection of piping using comb transducers. Mater. Eval.

[25] Rose J.L., Pelts S., Quarry M. (1998). A comb transducer model for guided wave NDE. Ultrasonics.

[26] Zhu W., Rose J.L. (1999). Lamb wave generation and reception with time-delay periodic linear arrays: A BEM simulation and experimental study. IEEE Trans. Ultrason. Ferroelec. Freq. Control.

[27] Li J., Rose J.L. (2001). Implementing guided wave mode control by use of a phased transducer array. IEEE Trans. Ultrason. Ferroelectr. Freq. Control.

[28] Giurgiutiu V. Lamb wave generation with piezoelectric wafer active sensors for structural health monitoring.

[29] Nieuwenhuis J., Neumann J., Greve D., Oppenheim I. (2005). Generation and detection of guided waves using PZT wafer transducers. IEEE Trans. Ultrason. Ferroelectr. Freq. Control.

[30] Jhang K.Y. (2009). Nonlinear ultrasonic techniques for non-linear destructive assessment of micro damage in material: A review. Int. J. Precis. Eng. Manuf..

[31] Sutin A. (1996). Nonlinear Acoustic Nondestructive Testing of Cracks. J. Acoust. Soc. Am..

[32] WaveFormRevealer software..

[33] Shen Y., Giurgiutiu V. WFR-2D: An analytical model for PWAS-generated 2D ultrasonic guided wave propagation.

[34] Biwa S., Hiraiwa S., Matsumotoa E. (2006). Experimental and theoretical study of harmonic generation at contacting interface. Ultrasonics.

[35] Shen Y., Giurgiutiu V. Health Monitoring of Aerospace Bolted Lap Joints Using Nonlinear Ultrasonic Spectroscopy: Theory and Experiments.

